# Phagocytosis of Cholesteryl Ester Is Amplified in Diabetic Mouse Macrophages and Is Largely Mediated by CD36 and SR-A

**DOI:** 10.1371/journal.pone.0000511

**Published:** 2007-06-06

**Authors:** Christopher B. Guest, Matthew E. Hartman, Jason C. O'Connor, Kenneth S. Chakour, Ali A. Sovari, Gregory G. Freund

**Affiliations:** 1 Division of Nutritional Sciences, University of Illinois at Urbana-Champaign, Urbana, Illinois, United States of America; 2 Department of Animal Sciences, University of Illinois at Urbana-Champaign, Urbana, Illinois, United States of America; 3 Department of Pathology, University of Illinois at Urbana-Champaign, Urbana, Illinois, United States of America; 4 College of Medicine, University of Illinois at Urbana-Champaign, Urbana, Illinois, United States of America; University of California, Merced, United States of America

## Abstract

Type 2 diabetes (T2D) is associated with accelerated atherosclerosis, which accounts for approximately 75% of all diabetes-related deaths. Here we investigate the link between diabetes and macrophage cholesteryl ester accumulation. When diabetic (*db/db*) mice are given cholesteryl ester intraperitoneally (IP), peritoneal macrophages (PerMΦs) recovered from these animals showed a 58% increase in intracellular cholesteryl ester accumulation over PerMΦs from heterozygote control (*db*/+) mice. Notably, PerMΦ fluid-phase endocytosis and large particle phagocytosis was equivalent in *db*/+and *db/db* mice. However, IP administration of CD36 and SR-A blocking antibodies led to 37% and 25% reductions in cholesteryl ester accumulation in PerMΦ. Finally, in order to determine if these scavenger receptors (SRs) were part of the mechanism responsible for the increased accumulation of cholesteryl esters observed in the diabetic mouse macrophages, receptor expression was quantified by flow cytometry. Importantly, *db/db* PerMΦs showed a 43% increase in CD36 expression and an 80% increase in SR-A expression. Taken together, these data indicate that direct cholesteryl ester accumulation in mouse macrophages is mediated by CD36 and SR-A, and the magnitude of accumulation is increased in *db/db* macrophages due to increased scavenger receptor expression.

## Introduction

T2D is an independent risk factor for developing atherosclerosis [1]. Accelerated atherosclerosis accounts for the majority of all diabetes-related deaths [2]. In addition, patients with T2D are 2 to 4 times more likely to develop atherosclerosis [3]. The incidence of individuals with diabetes who suffer a myocardial infarction is 20.2% compared to 3.5% for those without diabetes, following a second heart attack these numbers substantially increase to 45.0% and 18.8%, respectively [4]. Dyslipidemia, hypertension, oxidation state, endothelial cell function, hyperglycemia, insulin resistance, and advanced glycation end-products (AGEs) have been shown to play a role in diabetes-accelerated atherosclerosis [5] [6] [7]. However, it has not been determined if direct cholesteryl ester uptake by macrophages and subsequent foam cell formation may be altered in the diabetic state contributing to diabetes-accelerated atherosclerosis.

The storage of cholesteryl esters in macrophages and the subsequent formation of foam cells are critical to the development of the atherosclerotic plaque [8]. Both cholesteryl ester-rich oily droplets and cholesterol-rich vesicles are found within the early fatty streak and lipid core, with cholesteryl esters more prominent in the fatty streak and advanced plaques [9] [10]. Macrophages have been shown to directly take up both free cholesterol [11] and cholesteryl esters [12] through an as yet incompletely defined mechanism, resulting in the generation of foam cells. Initially in macrophage-derived foam cell development, monocytes migrate into the arterial intima stimulated by any or all of the following: tumor necrosis factor α, interleukin-1, 6, 8, 10, 12, oxidized LDL, monocyte chemoattractant protein 1-5, macrophage colony-stimulating factor, granulocyte/macrophage colony-stimulating factor, migratory inflammatory protein-1, transforming growth factor-β, RANTES, and endothelin-1 [13]. These monocytes then differentiate into macrophages and take up modified LDLs [13], which leads to the formation of foam cells through the accumulation of cholesteryl esters. LDLs are transported into lysosomes, where cholesteryl esters are hydrolyzed by acid hydrolase into free cholesterol. Cholesteryl ester repeatedly undergoes hydrolysis and re-esterification in the “cholesterol ester cycle” by neutral cholesterol ester hydrolase and acyl coenzyme A:cholesterol acyltransferase (ACAT), respectively [14]. Cholesterol in this process turns over with a half time of approximately 24 h [15]. The free cholesterol is exported from the cell or trapped in the cytosol as cholesteryl esters. Eventually, the accumulation of free cholesterol is toxic to the foam cell resulting in apoptosis [16] or necrosis [13], which contributes to the formation of the lipid core of the atherosclerotic plaque [17]. Tangirala et al. demonstrated that this same lysosomal accumulation of cholesterol occurs in macrophages loaded with cholesteryl esters through direct cholesteryl ester accumulation [18]. Furthermore, foam cells can proliferate within the atherosclerotic lesion [19] and activated macrophages can produce growth regulatory molecules, cytokines, and chemotactic factors [20], leading to atheroma progression.

SRs are integral to the development of foam cells in that they mediate the uptake of modified LDLs by macrophages [21] [22]. SRs were initially identified by Goldstein et al. with the discovery that macrophages take up acetylated LDL through a specific surface binding site that does not recognize native LDL [23]. Currently the scavenger receptor family consists of 6 structurally distinct classes of receptors (classes A through F) that bind a range of polyanionic ligands including modified LDLs [22]. Scavenger receptor A (SR-A) or CD204, and CD36, a class B member, have been shown to play a major role in the uptake of modified LDLs [24] [25]. Stangl et al. extended the role of scavenger receptors to include SR-BI mediated cholesteryl ester formation in the presence of free cholesterol [26], indicating that the scavenger receptor system plays a role in direct cholesterol uptake. Liang et al. further characterized the relationship between scavenger receptors and uptake of modified LDLs in a model of diabetes. This group demonstrated that due to defective insulin signaling, but independent of glucose and free fatty acids, macrophages from *ob/ob* mice express higher levels of SR-A and CD36 resulting in elevated cellular association with acetylated and oxidized LDL and increased cholesteryl ester formation [7]. In addition, reversal of insulin resistance in *ob/ob* mice with rosiglitazone, a peroxisome proliferator-activated receptor (PPAR) γ agonist, leads to decreased CD36 expression on PerMΦs [7]. Importantly, patients with T2D have increased CD36 expression on monocytes [27]. These studies demonstrate a plausible role for altered expression of scavenger receptors, specifically CD36 and SR-A in diabetes-associated atherosclerosis.

In summary, patients with T2D patients have an increased risk for developing atherosclerosis and the direct uptake of cholesteryl esters by macrophages following initiation of plaque development [9] [10] represents an important mechanism for foam cell formation [12]. However, the impact of diabetes on direct cholesteryl ester uptake by macrophages, which may play a role in plaque progression and perpetuation, has not been investigated. Therefore, we examined the hypothesis that macrophages from type-2 diabetic (*db/db*) mice have increased direct cholesteryl ester uptake and that this is mediated by CD36 and SR-A.

## Materials and Methods

### Materials

All cell culture reagents and chemicals were purchased from Sigma (St. Louis, MO) except as noted below. Lipoprotein (LDL) (cat# L-5402) and Lippoprotein (HDL) (cat#L-1567) were purchased from Sigma (St. Louis, MO). FBS (0.05 ng/ml, 0.48 EU/ml endotoxin) was purchased from Atlanta Biologicals (Norcross, GA). Bodipy-cholesteryl ester (cat# C-3927), Dil-acetylated LDL (cat# L-3484) and propidium iodide were purchased from Molecular Probes (Eugene, OR). TriColor-anti-mouse CD11b (cat# RM2806) and TriColor-rat IgG2b (cat# R2b06) were purchased from Caltag (Hamburg, Germany). Phycoerytherin-anti-mouse CD86 (cat# MCA1587PE), Phycoerytherin-rat IgG2a (cat# MCA1212PE), anti-mouse CD204 (SR-A) (cat# MCA1322), FITC-anti-mouse CD204 (SR-A) (cat# MCA1322F), and rat IgG2b (cat# MCA1125R) were purchased from Serotec (Raleigh, NC). 0.21 µm and 2.6 µm microspheres were purchased from Bangs Laboratories, Inc (Fishers, IN). Anti-mouse CD36 (cat# 552554), FITC-anti-mouse IgA (cat# 559354), FITC-rat-IgG2b (cat# 556923), and mouse IgA (cat# 553476) were purchased from Pharmingen, BD Biosciences (San Diego, CA). Anti-SR-BI (cat# NB 400-113 and NB 400-104E2) and FITC-anti-rabbit IgG (cat# NB 730-F) were purchased from Novus Biologicals (Littleton, CO). One Touch Ultra glucometer and glucose strips were purchased from Lifescan, Johnson&Johnson (Milpitas, CA). Sensitive Rat Insulin radioimmunoassay kit was purchased from Linco Research, Inc. (St. Charles, MO).

### Animals

All animal care and use was conducted in accordance with the Guide for the Care and Use of Laboratory Animals (NRC). 8- to 12-week-old B6.Cg-*M*+/+*Lepr* ^db^ (*db*/+) and B6.Cg-+*Lepr* ^db^/+*Lepr* ^db^ (*db/db*) were bred in house from mice purchased from The Jackson Laboratories (Bar Harbor, Maine). Mice were housed in standard shoebox cages and allowed pelleted food (NIH 5K52; LabDiet, Purina Mills Inc., Brentwood, MO) and water *ad libitum* in a temperature (72°C) and humidity (45–55%) controlled environment with a 12/12-h dark-light cycle (7:00 a.m.–7:00 p.m.).

### Peritoneal Macrophage Isolation

Mice were sacrificed by CO_2_ asphyxiation and peritoneal fluid was collected by lavaging the peritoneums twice with 5 ml ice cold low glucose growth media (glucose-free RPMI 1640 media supplemented with 10% FBS, 1 g/L glucose, 2 g/L sodium bicarbonate, 110 mg/L sodium pyruvate, 62.1 mg/L penicillin and 100 mg/L streptomycin, 10 mM HEPES pH 7.4), followed immediately by analysis or use in *ex vivo* experiments. For *ex vivo* experiments, cells were plated according to the following procedure. Peritoneal fluid was centrifuged and the resulting pellet resuspended in 5 ml of red blood cell lysis buffer (142 mM NaCl, 118 mM NaEDTA, 1 mM KHCO_3_ pH 7.4) at room temperature for 4 minutes. An equal volume of cold low glucose growth media was added followed by cell pelleting and resuspension in 37°C low glucose growth media. Cells were counted with the use of a hemocytometer and plated in culture dishes at 5×10^5^ cells/ml in low glucose growth media. After 30 min, plates were washed twice to remove non-adherent cells, resulting in approximately 80% pure macrophages, confirmed by CD11b staining and morphology [28]. Immediately following plating selection, peritoneal macrophages were used for Dil-AcLDL or bodipy-cholesteryl ester uptake experiments.

### Blood Glucose and Serum Insulin Measurement

Blood was collected from the lateral saphenous vein of 8-week-old *db*/+and *db/db* mice. Blood glucose levels were measured during collection using a One Touch Ultra® glucometer per the manufacturer's instructions. For random and fasting blood glucose measurements, blood was collected at 9:00 a.m. from mice fed ad libitum or fasted overnight, respectively. Serum insulin levels were measured by RIA according to the manufacturer's instructions. For random serum and fasting, blood was collected as above and serum was collected by centrifuging blood for 10 min at 16,000×g and stored at −20°C.

### CD11b/86 and CD36/SR-A Staining

After indicated treatments, PerMΦs were harvested as described above. Flow cytometry was performed as previously described [29]. In brief, cells were washed once in wash buffer (Dulbecco's phosphate-buffered saline (DPBS) containing 0.5% BSA without calcium and magnesium). TriColor, Phycoerytherin and/or FITC-conjugated antibodies at 10 µg/ml/test were added to 1×10^6^ cells in 100 µl of wash buffer then incubated on ice for 15 min followed by washing with wash buffer. Fluorescence was detected on an Epics XL flow cytometer (Beckman Coulter, Fullerton, CA). Gates were set to include CD11b and CD86 double positive cells and to exclude nonviable cells as determined by propidium iodide staining.

### Cholesterol and Particle Uptake

Cholesteryl ester labeled 1:1 with an ester bodipy tag, was dissolved in MeOH at 0.5 mg/ml by sonication for 60 min followed by sonication for 15 min prior to administration. Microscopic imaging studies of 5 µg/ml cholesteryl ester in sterile wash buffer demonstrated a non-homogeneous population localized to the coverslip, indicating that cholesteryl ester forms aggregates in a hydrophilic solution (Laboratory of Fluorescence Dynamics, University of Illinois, Urbana IL). Size was determined to range from approximately 1 µm to 3.5 µm (data not shown). For *ex vivo* particle uptake, cells were treated as indicated and allowed to incubate for 4 h at 37°C with 5 µg/ml bodipy-cholesteryl ester and stained with propidium iodide as above. To control for non-specific uptake, cells were treated as above and incubated for 4 h on ice with 5 µg/ml bodipy-cholesteryl ester. For *in vivo* uptake, mice were given an IP injection of 1 ml sterile filtered wash buffer containing either 5 µg/ml bodipy-cholesteryl ester, 5 µg/ml Dil-AcLDL, 5×10^7^ 2.6 µm beads, 5×10^9^ 0.21 µm beads, or 0.25 mg/ml FITC-dextran as indicated. After 4 h or as indicated, PerMΦs were harvested as described above without re-exposure to bodipy-cholesteryl ester *ex vivo*. Fluorescence was detected on an Epics XL flow cytometer (Beckman Coulter, Fullerton, CA). Gates were set to exclude nonviable cells as determined by propidium iodide staining. Cholesteryl ester uptake was quantified as previously described [30]; in brief, samples were excited at 490 nm and fluorescence was measured at 530 nm (554 and 571 for Dil-AcLDL), uptake was quantified by subtracting median fluorescence of control cells from median fluorescence of cells treated with bodipy-cholesteryl ester or Dil-AcLDL.

### CD36/SR-A, LDL/HDL Blocking Experiments

*In vivo* cholesterol uptake was performed as above. Mice were given an IP injection of 1 ml sterile filtered wash buffer containing 5 µg/ml bodipy-cholesteryl ester with 5 µg/ml of either anti-CD36, anti-SR-A, isotype control, 20 µg/ml LDL or 20 µg/ml HDL. After the times indicated, PerMΦs were harvested as described above. *Ex vivo* AcLDL uptake was performed as above for *ex vivo* cholesteryl ester uptake. Cells were incubated with 10 µg/ml of either anti-CD36, anti-SR-A, or isotype control for 15 m on ice. 5 µg/ml of Dil-AcLDL was added and cells incubated for 4 h. Fluorescence was detected as above. Gates were set to exclude nonviable cells as determined by propidium iodide staining. Median values of each population were used to indicate the level of cholesteryl ester uptake or AcLDL.

### Statistical Analysis

Where indicated, experimental data were analyzed either by the Student's t-test for comparison of means, or by ANOVA using Excel (Microsoft, Redmond WA). Statistical significance was denoted at p<0.05. Standard-error-of-the-mean bars are not shown when the error range is smaller than the symbol size.

## Results

### PerMΦs from db/db mice have increased AcLDL and cholesteryl ester uptake

**Figure 1:**
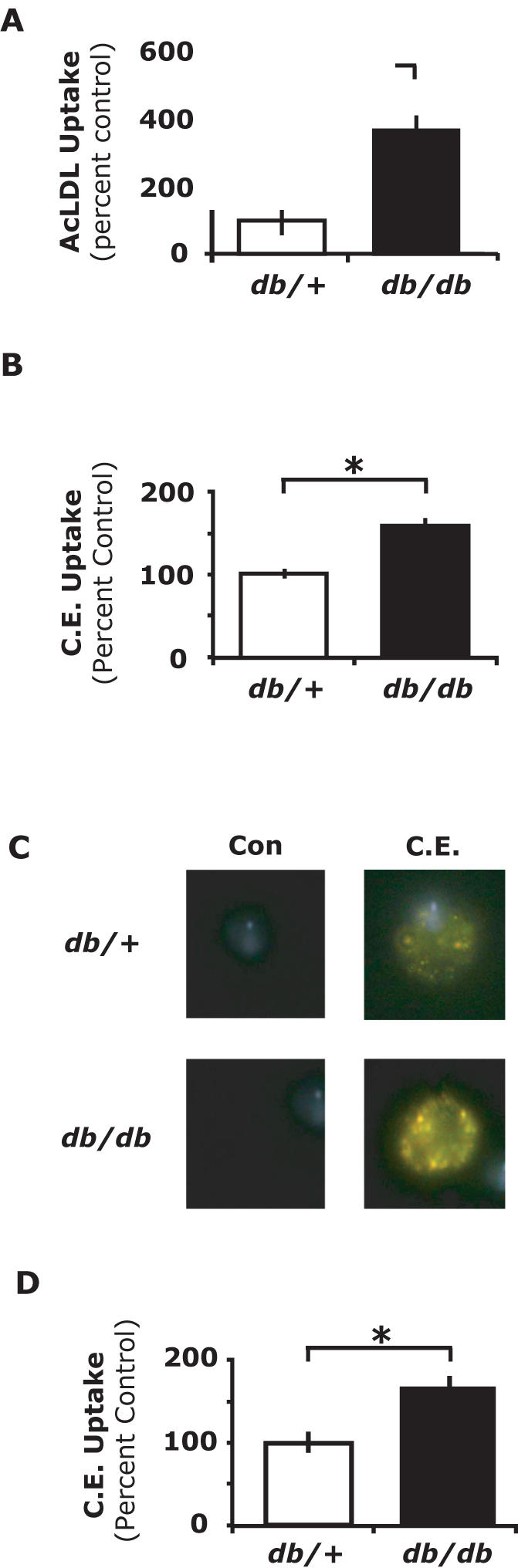
PerMΦs from *db/db* mice have increased AcLDL and cholesteryl ester uptake. *A, db*/+and *db/db* mice were administered 5 µg/mL of Dil-AcLDL by IP injection. After 4 h, PerMΦs were collected and Dil-AcLDL uptake measured by flow cytometry. *B*, db/+and db/db mice were administered 5 µg/mL of bodipy-cholesteryl ester by IP injection. After 4 h, PerMΦs were collected and cholesteryl ester uptake measured by flow cytometry. *C*, bodipy-cholesteryl ester (C.E) or carrier (Con) were administered as in A for 4 h. PerMΦs cholesteryl ester uptake was examined using fluorescent microscopy. Results are representative of three independent experiments. *D*, PerMΦs were collected from db/+and db/db mice and cultured for 30 min ex vivo. Cells were then incubated with 5 µg/mL bodipy-cholesteryl ester (C.E.) and cholesteryl ester uptake measured by flow cytometry at 4 h. Results for A, B and D represent the average of three independent experiments±SEM. *: p<0.05.

To determine if AcLDL or cholesteryl ester uptake was affected by diabetes, we examined resident PerMΦs from *db/db* and *db*/+mice. *Db/db* mice are characterized by hyperphagia, obesity, hyperinsulinemia and hyperglycemia (Table 1). One potential mechanism for the increased accumulation of lipids by diabetic MΦs is increased uptake of modified LDL. When Dil-AcLDL was administered IP, AcLDL uptake by PerMΦs *in vivo* was increased by 256% in *db/db* mice compared to *db*/+mice (p<0.001) (Fig. 1A). Next, direct uptake of cholesteryl ester was measured. Fig. 1B shows that when bodipy-cholesteryl ester was administered IP for 4 h, cholesteryl ester uptake by PerMΦs *in vivo* was increased by 58% in *db/db* mice compared to *db*/+mice (p<0.01). Importantly, intracellular localization of bodipy-cholesteryl ester was confirmed by fluorescent microscopy (Fig. 1C). To ascertain if PerMΦs from *db/db* mice had increased cholesteryl ester uptake that required the involvement of a peritoneal factor, PerMΦs isolated and cultured from *db/db* and *db*/+mice were examined *ex vivo*. Fig. 1D shows that, when cultured PerMΦs from *db/db* and *db*/+mice were incubated with bodipy-cholesteryl ester for 4 h, cholesteryl ester uptake was increased by 59% (p<0.01) in *db/db* mouse PerMΦs. These results indicate that PerMΦs from *db/db* mice have increased ability to take up AcLDL *in vivo* and cholesteryl esters both *in vivo* and *ex vivo*.

**Table 1:** *Db/db* mice have elevated blood glucose and serum insulin levels.

	*db*/+	*db/db*
**Body Wt (g)**	**27.28±0.79**	38.88±0.84*
**FBG (mg/dl)**	**95.6±10.57**	408.8±52.77*
**RBG (mg/dl)**	**154.6±14.03**	434.0±50.88*
**FSI (ng/ml)**	**1.36±0.23**	2.36±0.045*
**RSI (ng/ml)**	**2.04±0.48 (0.356 nM)**	5.34±0.11* (0.932 nM)

Fasting blood glucose (FBG), random blood glucose (RBG), fasting serum insulin (FSI), and random serum insulin (RSI). Results represent the average of n = 5 mice±SEM. *: p<0.05.

### Large particle phagocytosis and fluid-phase endocytosis are not increased in PerMΦs from db/db mice

**Figure 2:**
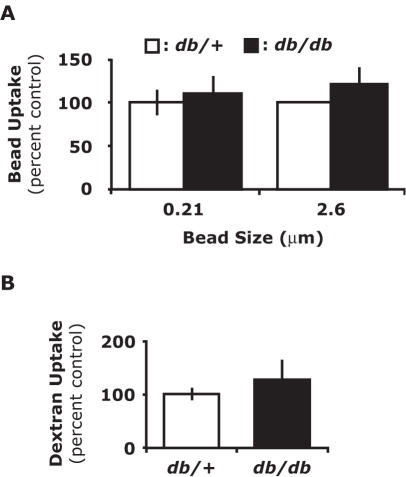
Phagocytosis and fluid-phase endocytosis are not increased in PerMΦs from *db/db* mice. *A*, *Db*/+and *db/db* mice were IP administered 0.21 or 2.6 µm latex microspheres (beads) at 5×10^9^ or 5×10^7^ beads/ml, respectively. Following a 4 h *in vivo* incubation period, PerMΦs were collected and microsphere uptake was measured by flow cytometry. *B, Db*/+and *db/db* mice were IP administered 0.25 mg/ml FITC-dextran. Following a 4 h *in vivo* incubation period, PerMΦs were collected and dextran uptake was measured by flow cytometry. Results for *A* and *B* represent the average of three independent experiments±SEM.

The mechanism by which cholesteryl esters are taken up by macrophages is not well defined. Cholesteryl esters can enter the cell via receptor-mediated endocytosis or alternatively, cholesteryl esters in solution may form microcrystals [31] that can be ingested by phagocytosis or fluid-phase endocytosis. To determine if phagocytosis was increased in *db/db* PerMΦs, we examined 0.21 µm microsphere and 2.6 µm microsphere uptake [32]. Fig. 2A shows that PerMΦs from *db/db* mice did not have increased microsphere uptake when compared to PerMΦs from *db*/+mice (0.21 µm, p = 0.609; 2.6 µm, p = 0.260). Likewise, we examined fluid-phase endocytosis in *db/db* PerMΦs utilizing dextran [32]. Fig. 2B demonstrates that PerMΦs from *db/db* mice did not have increased uptake of dextran over PerMΦs from *db*/+mice (p = 0.450). These results indicate that large particle phagocytosis and fluid-phase endocytosis are not increased in *db/db* mouse PerMΦs.

### CD36 and SR-A mediate amplified AcLDL and cholesteryl ester uptake in db/db PerMΦs

**Figure 3:**
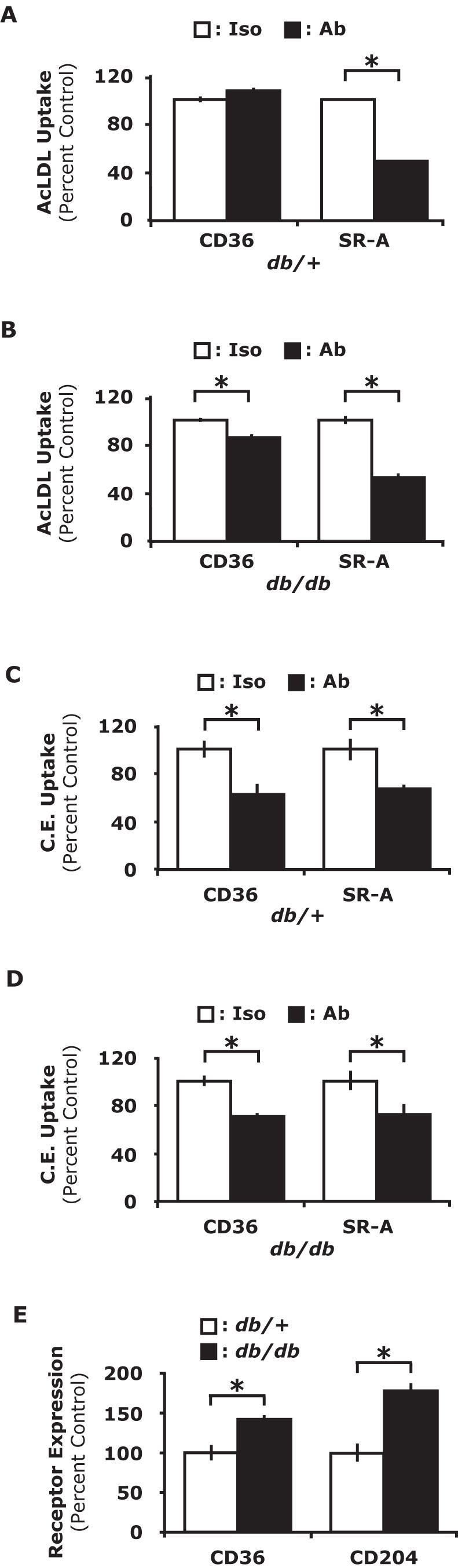
CD36 and SR-A mediate increased AcLDL and cholesteryl ester uptake in diabetic PerMΦs. *A,* PerMΦs were collected from *db*/+mice and cultured for 30 min ex vivo. Cells were then incubated on ice for 15 m with CD36, SR-A blocking antibodies (Ab) or isotype control antibodies (Iso) as indicated. Cells were then incubated with 5 µg/ml Dil-AcLDL and uptake measured by flow cytometry at 4 h. *B,* PerMΦs were collected from *db/db* mice and cultured for 30 min ex vivo. Cells were then incubated on ice for 15 m with CD36, SR-A blocking antibodies (Ab) or isotype control antibodies (Iso) as indicated. Cells were then incubated with 5 µg/ml Dil-AcLDL and uptake measured by flow cytometry at 4 h. *C, Db*/+mice were IP administered 5 µg/ml bodipy-cholesteryl ester (C.E.) with CD36, SR-A blocking antibodies (Ab) or isotype control antibodies (Iso) as indicated. After 4 h, PerMΦs were collected and cholesteryl ester uptake measured by flow cytometry. *D, Db/db* mice were IP adminsitered 5 µg/ml bodipy-cholesteryl ester (C.E.) with CD36, SR-A blocking antibodies (Ab) or isotype control antibodies (Iso) as indicated. After 4 h, PerMΦs were collected and cholesteryl ester uptake measured by flow cytometry. *E*, PerMΦs were collected from *db*/+and *db/db* mice. CD36 and SR-A surface expression was measured by flow cytometry. Results for *A-E* represent the average of three independent experiments±SEM. *: p<0.05

Macrophages take up modified LDLs through scavenger receptors leading to the formation of foam cells [8]. The class B scavenger receptor CD36 and the class A scavenger receptor SR-A are considered two of the most important for foam cell formation [21] and are increased in the presence of insulin resistance [7]. To determine the relative importance of CD36 and SR-A in the uptake of AcLDL in *db*/+and *db/db* PerMΦs *ex vivo* antibody-dependent blocking experiments were performed. Fig. 3A shows that when anti-CD36 was administered to PerMΦs from *db*/+mice, AcLDL uptake was not significantly different than isotype control (p = 0.06). As expected, administration of anti-SR-A blocked more than 50% of AcLDL uptake when compared to isotype control (p<0.0001). Next, we examined the effect of these antibodies on AcLDL uptake in *db/db* PerMΦs. Fig. 3B shows that anti-CD36 decreased AcLDL uptake by 15% (p<0.001). Similarly, as seen in *db*/+PerMΦs, anti-SR-A significantly reduced the uptake of AcLDL by 48% (p<0.0002). Taken together these results indicate that SR-A plays an important role in the uptake of AcLDL in both *db*/+and *db/db* PerMΦs, however, CD36 appears only to significantly impact AcLDL uptake in *db/db* PerMΦs. Next, the role of CD36 and SR-A in cholesteryl ester uptake was examined using *in vivo* antibody-dependent blocking of CD36 and SR-A. Fig. 3C shows that, when anti-CD36 and anti-SR-A were administered IP to *db*/+mice, *in vivo* PerMΦ cholesteryl ester uptake was reduced by 37% (p<0.01) and 25% (p<0.05) when compared to isotype controls, respectively, indicating that CD36 and SR-A mediate the uptake of cholesteryl ester. Similarly, when anti-CD36 and anti-SR-A were administered IP to *db/db* mice, *in vivo* PerMΦ cholesteryl ester uptake was reduced by 31% (p<0.01) and 28% (p<0.05), respectively (Fig. 3D). Finally, to determine if expression of CD36 and/or SR-A were increased in PerMΦs from *db/db* mice, flow cytometry was performed. Fig. 3E demonstrates that *db/db* mouse PerMΦs had a 43% increase in CD36 expression (p<0.01) and an 80% increase in SR-A expression when compared to PerMΦs from *db*/+mice (p<0.01). In order to determine if physiologically relevant concentrations of LDL or HDL mediated the uptake of cholesteryl esters, LDL or HDL were administered as indicated above. Co-administration of LDL or HDL failed to reduce the accumulation of cholesteryl ester (data not shown). Taken together these results indicate that increased CD36 and SR-A expression on *db/db* mouse PerMΦs is responsible for enhanced AcLDL and direct cholesteryl ester uptake observed in *db/db* mice.

## Discussion

Foam cell formation is central to the development and progression of the atherosclerotic plaque [13], [16], [17], [19], [20]. Key components to foam cell formation are macrophage ingestion and accumulation of cholesteryl esters via SR-mediated uptake of modified LDLs [8] and transport of cholesteryl esters across the membrane in conjunction with HDL and reverse cholesterol transport [33]. We found that PerMΦs from *db/db* mice have increased AcLDL uptake compared to *db*/+controls (Fig. 1A). Given the central role of SRs to this process, we conducted antibody blocking experiments to determine the relative importance of CD36 and SR-A in *db*/+and *db/db* PerMΦs. Antibody blocking experiments showed that anti-CD36 had little affect on AcLDL uptake in *db*/+PerMΦs while in *db/db* PerMΦs anti-CD36 attenuated AcLDL uptake 15%. This finding demonstrates an interesting phenotypic difference between *db*/+and *db/db* PerMΦs and may explain, in part, the significant increase of AcLDL uptake seen in *db/db* PerMΦs. Next, the role of SR-A in AcLDL was examined. The administration of anti-SR-A antibodies decreased AcLDL uptake by 50% in *db*/+PerMΦs and 48% in *db/db* PerMΦs indicating that the uptake of AcLDL is primarily mediated by SR-A rather than CD36 (Fig. 3A–B). These findings support work by Liang *et al.* which demonstrated that insulin resistance leads to increased cellular uptake with AcLDL [7], although, in a different mouse model of diabetes. Uptake of modified LDL by SRs and accumulation of CE is well characterized but the importance of SRs for direct CE uptake has yet to be investigated thoroughly investigated [26]. In addition to cholesterol deposition through lipoprotein metabolism, direct cholesterol and cholesteryl ester uptake has been demonstrated in macrophages [11], [12]. Currently it is uncertain whether diabetic macrophages have increased direct uptake of cholesteryl esters and how this may contribute to the pathogenesis of atherosclerosis. In Fig. 1B, we show that PerMΦs from *db/db* mice have increased uptake of cholesteryl esters at 4 hours post IP administration. McGookey *et al.* [15] measured the half-life of cholesteryl ester hydrolysis into cholesterol and fatty acid subunits to be approximately 24 hours. Importantly, the fluorescent bodipy label is conjugated to the fatty acyl moiety of the cholesteryl ester indicating that an increase in intracellular fluorescent bodipy label at 4 hours directly relates to an increase in intracellular cholesteryl ester and not label alone. We have also confirmed that the median fluorescence of the bodipy-cholesteryl ester is linearly related to the mass of cholesteryl ester (data not shown). To confirm that cholesteryl esters were taken into the cell and not adhered to the cell surface, fluorescent microscopy was performed. Fig. 1C demonstrates that cholesteryl esters were located intracellularly. It is possible that the cholesteryl ester injected IP formed liposomes with apoproteins present in the peritoneal fluid or interacted with other peritoneal factors that led to macrophage cholesteryl ester uptake. Fig. 1D, however, demonstrates that PerMΦs from *db*/+and *db/db* mice cultured *ex vivo* had a similar cholesteryl ester uptake profile as PerMΦs *in vivo*. Thus, PerMΦs directly take up cholesteryl esters independently from any intraperitoneal factors and in diabetes this uptake is increased. These findings are important because cholesteryl esters are found in fatty streaks and in the lipid core of atheromas [9] [10] thus plaque based macrophages in diabetes may utilize this cholesteryl ester reservoir more readily to form foam cells than non-diabetic macrophages.

The next question to address was how does the diabetic state lead to increased cholesteryl ester uptake by PerMΦs. Macrophages can ingest extracellular particles by receptor-mediated endocytosis, fluid-phase endocytosis and/or phagocytosis. In order to better define how diabetic macrophages ingest cholesteryl esters, large particle phagocytosis and fluid-phase endocytosis was examined via latex bead (Fig. 2A) and dextran (Fig. 2B) uptake, respectively. As shown in Fig. 2, neither of these mechanisms of ingestion differed between *db*/+and *db/db* mice. This indicated that increased cholesteryl ester uptake was likely mediated by receptor-mediated endocytosis. Mahlberg *et al.* demonstrated uptake of cholesteryl ester lipid droplets by a phagocytic process in J774 macrophages [12] and Stangl *et al.* demonstrated that overexpression of SR-BI led to an increase in nonlipoprotein cholesterol uptake by CHO cells [26]. Therefore, it seemed likely that scavenger receptors may be involved in the uptake of cholesteryl esters, especially SR-BI. However, SR-BI expression was not detectable in peritoneal macrophages from either *db*/+or *db/db* mice. SR-BI was detected on macrophage like RAW 264.7 cells (data not shown). Liang et al. recently demonstrated that insulin resistance leads to increased expression of CD36 and SR-A and cellular uptake of acetylated and oxidized LDL [7]. Our data in Fig. 1A&Fig. 3E supports these findings in a different animal model of diabetes. Additionally, we examined the relative contribution of CD36 and SR-A to the uptake of AcLDL in PerMΦs from *db*/+and *db/db* mice. Fig. 3A demonstrates that CD36 does not significantly contribute to the uptake of AcLDL in *db*/+PerMΦs. However, Fig. 3B shows that the administration of anti-CD36 specific blocking antibodies significantly decreased the uptake of AcLDL in *db/db* PerMΦs. Since the same concentration of anti-CD36 antibody was able to decrease the uptake of AcLDL in the *db/db* PerMΦs, it demonstrated that this antibody was effective as a blocking antibody. This finding may be an important phenotypic difference between the *db*/+and the *db/db* PerMΦs, not only with respect to modified LDL uptake, but it may impact the innate immune response to ligands that interact with Toll-like-receptor 2 (TLR-2), since CD36 has been identified as costimulatory molecule for this TLR-2 [34]. Fig. 3C–D extends these finding to show that CD36 and SR-A were linked to direct cholesteryl ester uptake in PerMΦs. Furthermore, since both of these SRs are involved in direct cholesteryl ester uptake, increased expression of CD36 and SR-A observed on *db/db* mouse PerMΦs (Fig. 3E) appears responsible for their enhanced cholesteryl ester uptake by increasing the magnitude of uptake.

Lastly, the mechanism by which scavenger receptors mediate direct cholesteryl ester uptake has not been delineated. One possibility is that the cholesteryl esters interact with LDL or HDL in some fashion that then results in uptake through these already established mechanisms. However, neither co-administration of LDL nor HDL was able to reduce IP cholesteryl ester uptake by peritoneal macrophages (data not shown). This is not surprising given work done by Goldstein et al. demonstrating that unmodified LDL does not interact strongly with the scavenger receptor system [23] and that the HDL receptor, SR-BI, was not detected on PerMΦs. Therefore, it appears that the scavenger receptors CD36 and SR-A mediate direct uptake of cholesteryl esters by macrophages through a mechanism distinct from LDL and HDL uptake. Although at this time more work is required to define the exact mechanism of direct cholesteryl ester uptake, our data here demonstrate that the diabetic state produces an increase in direct cholesteryl ester uptake by PerMΦs, a finding that may prove significant in continuing the search to delineate the pathogenesis of diabetes-accelerated atherosclerosis.

## References

[1] Aronson D, Rayfield EJ (2002). How hyperglycemia promotes atherosclerosis: Molecular mechanisms.. Cardiovasc Diabetol.

[2] Hurst RT, Lee RW (2003). Increased incidence of coronary atherosclerosis in type 2 diabetes mellitus: Mechanisms and management.. Ann Intern Med.

[3] Williams G, Pickup JC (1999). Handbook of diabetes..

[4] Haffner SM, FAU-Lehto S, Lehto S, FAU-Ronnemaa T, Ronnemaa T (1998). Mortality from coronary heart disease in subjects with type 2 diabetes and in nondiabetic subjects with and without prior myocardial infarction.. -N Engl J Med..

[5] Askari B, Renard CB, Bornfeldt KE (2002). Regulation of smooth muscle cell accumulation in diabetes-accelerated atherosclerosis.. Histol Histopathol.

[6] Jagasia D, McNulty PH (2003). Diabetes mellitus and heart failure.. Congest Heart Fail.

[7] Liang CP, Han S, Okamoto H, Carnemolla R, Tabas I (2004). Increased CD36 protein as a response to defective insulin signaling in macrophages.. J Clin Invest.

[8] de Villiers WJ, Smart EJ (1999). Macrophage scavenger receptors and foam cell formation.. J Leukoc Biol.

[9] Katz SS, Shipley GG, Small DM (1976). Physical chemistry of the lipids of human atherosclerotic lesions. demonstration of a lesion intermediate between fatty streaks and advanced plaques.. J Clin Invest.

[10] Guyton JR, Klemp KF (1996). Development of the lipid-rich core in human atherosclerosis.. Arterioscler Thromb Vasc Biol.

[11] Lesnik P, Rouis M, Skarlatos S, Kruth HS, Chapman MJ (1992). Uptake of exogenous free cholesterol induces upregulation of tissue factor expression in human monocyte-derived macrophages.. Proc Natl Acad Sci U S A.

[12] Mahlberg FH, Glick JM, Jerome WG, Rothblat GH (1990). Metabolism of cholesteryl ester lipid droplets in a J774 macrophage foam cell model.. Biochim Biophys Acta.

[13] Takahashi K, Takeya M, Sakashita N (2002). Multifunctional roles of macrophages in the development and progression of atherosclerosis in humans and experimental animals.. Med Electron Microsc.

[14] Brown MS, Ho YK, Goldstein JL (1980). The cholesteryl ester cycle in macrophage foam cells. continual hydrolysis and re-esterification of cytoplasmic cholesteryl esters.. J Biol Chem.

[15] McGookey DJ, Anderson RG (1983). Morphological characterization of the cholesteryl ester cycle in cultured mouse macrophage foam cells.. J Cell Biol.

[16] Kellner-Weibel G, Jerome WG, Small DM, Warner GJ, Stoltenborg JK (1998). Effects of intracellular free cholesterol accumulation on macrophage viability: A model for foam cell death.. Arterioscler Thromb Vasc Biol.

[17] Ball RY, Stowers EC, Burton JH, Cary NR, Skepper JN (1995). Evidence that the death of macrophage foam cells contributes to the lipid core of atheroma.. Atherosclerosis.

[18] Tangirala RK, Mahlberg FH, Glick JM, Jerome WG, Rothblat GH (1993). Lysosomal accumulation of unesterified cholesterol in model macrophage foam cells.. J Biol Chem.

[19] Gordon D, Reidy MA, Benditt EP, Schwartz SM (1990). Cell proliferation in human coronary arteries.. Proc Natl Acad Sci U S A.

[20] Ross R (1995). Cell biology of atherosclerosis.. Annu Rev Physiol.

[21] Hiltunen TP, Yla-Herttuala S (1998). Expression of lipoprotein receptors in atherosclerotic lesions.. Atherosclerosis.

[22] Greaves DR, Gough PJ, Gordon S (1998). Recent progress in defining the role of scavenger receptors in lipid transport, atherosclerosis and host defence.. Curr Opin Lipidol.

[23] Goldstein JL, Ho YK, Basu SK, Brown MS (1979). Binding site on macrophages that mediates uptake and degradation of acetylated low density lipoprotein, producing massive cholesterol deposition.. Proc Natl Acad Sci U S A.

[24] Suzuki H, Kurihara Y, Takeya M, Kamada N, Kataoka M (1997). A role for macrophage scavenger receptors in atherosclerosis and susceptibility to infection.. Nature.

[25] Silverstein RL, Febbraio M (2000). CD36 and atherosclerosis.. Curr Opin Lipidol.

[26] Stangl H, Cao G, Wyne KL, Hobbs HH (1998). Scavenger receptor, class B, type I-dependent stimulation of cholesterol esterification by high density lipoproteins, low density lipoproteins, and nonlipoprotein cholesterol.. J Biol Chem.

[27] Sampson MJ, Davies IR, Braschi S, Ivory K, Hughes DA (2003). Increased expression of a scavenger receptor (CD36) in monocytes from subjects with type 2 diabetes.. Atherosclerosis.

[28] Ceddia MA, Woods JA (1999). Exercise suppresses macrophage antigen presentation.. J Appl Physiol.

[29] Deszo EL, Brake DK, Cengel KA, Kelley KW, Freund GG (2001). CD45 negatively regulates monocytic cell differentiation by inhibiting phorbol 12-myristate 13-acetate-dependent activation and tyrosine phosphorylation of protein kinase cdelta.. J Biol Chem.

[30] Epps DE, Harris JS, Greenlee KA, Fisher JF, Marschke CK (1995). Method for measuring the activities of cholesteryl ester transfer protein (lipid transfer protein).. Chem Phys Lipids.

[31] Snow JW, McCloskey HM, Glick JM, Rothblat GH, Phillips MC (1988). Physical state of cholesteryl esters deposited in cultured macrophages.. Biochemistry.

[32] Tyteca D, Van Der Smissen P, Mettlen M, Van Bambeke F, Tulkens PM (2002). Azithromycin, a lysosomotropic antibiotic, has distinct effects on fluid-phase and receptor-mediated endocytosis, but does not impair phagocytosis in J774 macrophages.. Exp Cell Res.

[33] Gargalovic P, Dory L (2003). Caveolins and macrophage lipid metabolism.. J Lipid Res.

[34] Hoebe K, FAU-Georgel P, Georgel P, FAU-Rutschmann S, Rutschmann S (2005). CD36 is a sensor of diacylglycerides.. -Nature.

